# Pathology and Treatment of Croup

**Published:** 1847-09

**Authors:** 


*Pathology and Treatment of Croup.* By Mr. Hird.—The author of this communication believes croup to be an inflammatory disease of a specific nature, and distinguishes it from the purely spasmodic closure of the glottis, which, in its commencement, it often closely resembles. He states that in examination of fatal cases, where the patients survived the attack only a few hours, and in which the inflammatory symptoms were the most severe, he observed only slight traces of albuminous exudation, the mucous membrane of the trachea and larynx presenting a red and swollen appearance, and being covered with a tenacious and sanguineous frothy mucus. When death occurred two or three days after the commencement of the attack, and where the inflammatory symptoms, although fully developed, were of a less acute character, a pseudo-membrane, of a grayish-yellow color, and about a line in thickness, occupied either the trachea only, or the trachea, larynx, and bronchial tubes. In some cases, a viscid muco-purulent secretion adhered to the mucous membranes of the air-tubes. In the air-cells and parenchyma of the lungs, a reddish serum was frequently found; hepatization, also, was occasionally observed. The croupy membrane, he stated, may adhere throughout its entire extent to the mucous membrane of the air-passages, or hang loose from a pedicle, like a polypus, or attached in the centre, and free at one or both extremities, or it may be completely separated from the mucous membrane by a muco-purulent secretion.

In the treatment of croup, Mr. Hird stated, that during the last four or five years he had never resorted to general blood-letting, and that he was induced to adopt this course, from witnessing the unfavorable result which generally followed its use, both in his own practice, and in that of some of his professional friends. Croup, he considers an inflammation of a character bearing some analogy to erysipelas, or to inflammation occurring in scrofulous individuals, which occasions the formation of a pseudo-membrane, that will require for its removal a certain amount of vital energy, both in the respiratory organs and in the system generally. The inflammation of croup he believes to be less under the influence of blood-letting than healthy inflammation, and that whether it be looked upon in reference to the alteration to be expected from it on the progress of croup, or on the subsequent effects of the evacuation on the system, it will be found both unsatisfactory and dangerous.

The class of cases in which he occasionally prescribes leeches, or a cupping-glass to the chest, are those acute affections in which there is not the same disposition to the formation of a croupy membrane, and in which the larynx is affected. The inflammation, he considers, approaches more to the character of healthy inflammation, is more under the control of blood-letting, and is apt to cause effusion near the glottis, which speedily proves fatal.

In the early stage of both varieties of the disease—i. e., both in the ordinary tracheal disease, as well as in the more acute affection in which the larynx is affected—he gives, in the first instance, an antimonial emetic, the dose varying from a quarter of a grain to a grain, according to the age of the child. After vomiting has been produced, he orders three grains of calomel, and if necessary, repeats the dose, that the bowels may be freely acted upon. If, by these measures, the febrile and inflammatory symptoms are in some degree subdued, he finds the greatest benefit from the free use of the alkalies. So long, however, as the fever continues unabated, and the heart's action unsubdued, he keeps up the state of nausea with a solution of the potassio-tartrate of antimony, in doses varying from a twelfth to a quarter of a grain every half-hour or hour, until a decided check to the symptoms is produced. As a local application to the throat, he advises a flannel bag half filled with hot salt, as recommended by Mr. Kirby, of Dublin. The warm bath, and a blister between the shoulders, or on the sternum, for an hour or two, are often of great assistance. Mr. Hird considers that the alkalies act by allaying the irritation which produces the paroxysms of spasm, in the same manner as they allay the cough in hooping-cough; and that they are as valuable in promoting the absorption of the albuminous exudation thrown out in this disease, as they have been proved to be by Sir B. Brodie, in the removal of large fatty tumors occuring in various parts of the body. He prescribes ten or fifteen minims of the liquor potassæ every four hours, or in smaller doses more frequently repeated. At a later period of the disease, he gives the decoction of senega, combined with ipecacuanha, squills, and ammonia.—*London Lancet.*—*Rankin's Abstract.*

